# Wood smoke fire particulate matter induces eosinophilic asthma in a Sprouty2-dependent manner in mice

**DOI:** 10.3389/falgy.2026.1951459

**Published:** 2026-10-30

**Authors:** Anand Sripada, Rangati Varma, Kapil Sirohi, Arnav Gupta, Anthony N. Gerber, Rafeul Alam

**Affiliations:** 1 The Division of Allergy and Immunology, Department of Medicine, National Jewish Health, Denver, CO, United States; 2 Immunology and the Division of Pulmonary and Critical Care Medicine, Department of Medicine, National Jewish Health, Denver, CO, United States; 3 Division of Pulmonary, Critical Care and Sleep Medicine, University of Kentucky, Lexington, KY, United States

**Keywords:** airway hyperreactivity, asthma, eosinophils, innate lymphoid cell (ILC), wood smoke particles

## Abstract

Wood smoke particulate matter (WSP) is an important public health hazard. The objective of this study was to establish a mouse model of WSP-induced airway inflammation and hyperreactivity. WSP has been linked to a genetic risk variant that regulates the expression of the ERK1/2 regulator Sprouty2 (Spry2). We examined the importance of Spry2 in WSP-induced airway pathology and activation of critical immune cells in mice with inducible Spry2 gene deletion. A short-term airway exposure of naïve B6 mice to WSP induced airway hyperreactivity to methacholine and eosinophilic, and, to a lower extent, modest neutrophilic inflammation, mucus production and airway remodeling. The induction of the foregoing pathologic abnormalities was associated with number of ILC2s and CD4 T cells, increased ERK1/2 phosphorylation, and increased production of IL5, IL13 and IL17A. Inducible deletion of Spry2 prevented the induction airway hyperreactivity, airway inflammation, mucus production, Collagen deposition, and phosphorylation of ERK1/2. Spry2-/- inhibited the increase in ILC2 but not CD4 T cell frequency. ERK1/2 phosphorylation was inhibited in both ILC2s and Th2 cells in the absence of Spry2. Spry2 deletion reduced IL5, IL13 and IL17A expressing ILC2s. Similarly, Spry2 deletion reduced production of IL17A and IL13 but not IL5 in CD4 T cells. We conclude that WSP induces eosinophilic and modestly neutrophilic airway inflammation and other features of asthma. Spry2 plays a non-redundant role in WSP-induced airway pathologies, in part, by facilitating ERK1/2 activation.

## Introduction

Wildfires, driven by the impacts of climate change and legacy forest management strategies, are becoming a major global public health concern (1, 2). Wood smoke particulate matter (WSP) is a complex mixture of air pollutants, including significant amounts of fine particulate matter [i.e., with an aerodynamic diameter ≤2.5 μm (PM_2.5_)], carbon oxides, nitrogen oxides, hazardous air pollutants, water vapor, and trace levels of several other compounds (3). Exposure to WSP is associated with an increased risk of respiratory illnesses including, impaired lung function and exacerbations in asthma, chronic obstructive pulmonary disease, and respiratory infection (1, 4, 5). Furthermore, studies have shown a 27% increase in hospital and emergency department visits for respiratory complications as a consequence from wildfire smoke-derived PM_2.5_ (6). Recent findings suggest genetic factors that may contribute to asthma risk in the context of WSP exposure and identified Sprouty2 (Spry2) as a mediator that facilitates asthma risk in response to wildfire PM_2.5_ (7).

Sprouty (Spry) proteins were initially discovered in *Drosophila melanogaster* as an inhibitor of fibroblast growth factor (FGF)-induced tracheal branching (8). Spry proteins are ubiquitously expressed and at least 4 mammalian Spry orthologs (Spry1 to 4) have been identified, of which Spry3 expression is restricted to brain and testis (9–11). Subsequent work on Spry protein has demonstrated its role in inhibition of the RTK/Ras/ERK pathway downstream of several growth factors (12). This general inhibitory function of Spry is both cell and ligand specific, because of its ability to interact with key signaling molecules in the Ras/ERK1/2 signaling pathway such as Grb2, Shp2, Raf, Sos1, TESK, and FRS2 (13–15). On the other hand, Spry2 is also known to enhance ERK activation, by preventing c-cbl mediated degradation of EGF receptor (16). We have recently shown a positive regulatory role for Spry2 in TCR-triggered ERK1/2 phosphorylation, where loss of Spry2 leads to reduced ERK phosphorylation in T-cells (17). Taken together, these results suggest that Spry2, depending on the context and cell type, could function either as a positive- or a negative regulator of ERK1/2 activation.

ERK1/2 signaling plays a major role in Th2 differentiation and allergic airway inflammation (18, 19). Our lab has previously shown that Spry2 deficiency leads to defective ERK phosphorylation in CD4^+^ T-cells and attenuated allergic airway inflammation in response to allergens (*Aspergillus fumigatus*) (17). With this background we sought to investigate whether Spry2 played a role in WSP-induced airway inflammation, and whether loss of Spry2 leads to reduced ERK1/2 phosphorylation in immune cells exposed to WSP. We present evidence that Spry2 deficiency leads to attenuated airway hyperreactivity (AHR), reduced lung inflammation and defective ERK1/2 phosphorylation in CD4^+^ T-cells and in innate lymphoid cells (ILCs).

## Materials and methods

### Mice

*Spry2^+/+^* and *Spry2^−/–^* mice were generated as previously described (17). Briefly, mice with Cre recombinase-ERT2 fusion gene driven by ROSA26 promotor (B6.129-Gt(ROSA)26Sor^tm1(Cre/ERT2)Tyj^/J from The Jackson's Laboratory) and *Spry2^f/f^*[mutant mice research and resource center (MMRRC)] were crossed to generate mice carrying ERT2-Cre: *Spry2^f/f^*. Global deletion of Spry2 (*Spry2^−/−^*) was accomplished by intraperitoneal injection of 100 *μ*l of 10 mg/mL Tamoxifen (Sigma, cat. no. T5648) for 5 consecutive days into 4-wk-old ERT2-Cre: *Spry2^f/f^* mice. Similarly, Tamoxifen treated sex- and age-matched *Spry2^f/f^* mice were used as controls (*Spry2^+/+^*) in all our experiments. After Tamoxifen treatment, mice were rested for at least 3 wk before use in experiments. 8- to 12- wk old female mice were used in this study and the animals were maintained according to the National Jewish Health IACUC guidelines (Protocol no. AS2614-11-23).

### Animal model

We used WSPs that were generated as described and generously gifted by Dr Andrew Ghio at the Environmental Protection Agency Laboratory in North Carolina (20). Wood smoke was generated by heating white oak wood on an electric heating element (Brinkmann Corporation) in a Quadra-fire 3,100 woodstove (Colville). The mean diameter of the freshly generated WSP was 0.14 micron. For the WSP-driven acute asthma model, female *Spry2^+/+^* and *Spry2^−/−^* mice were intranasally challenged with WSP (25 µg) for 5 consecutive days in the first week and on alternate days in the second week as shown in the Figure 1B. Two days after the last challenge, mice were examined for AHR, lung infiltrating immune cells and lung inflammation.

**Figure 1:**
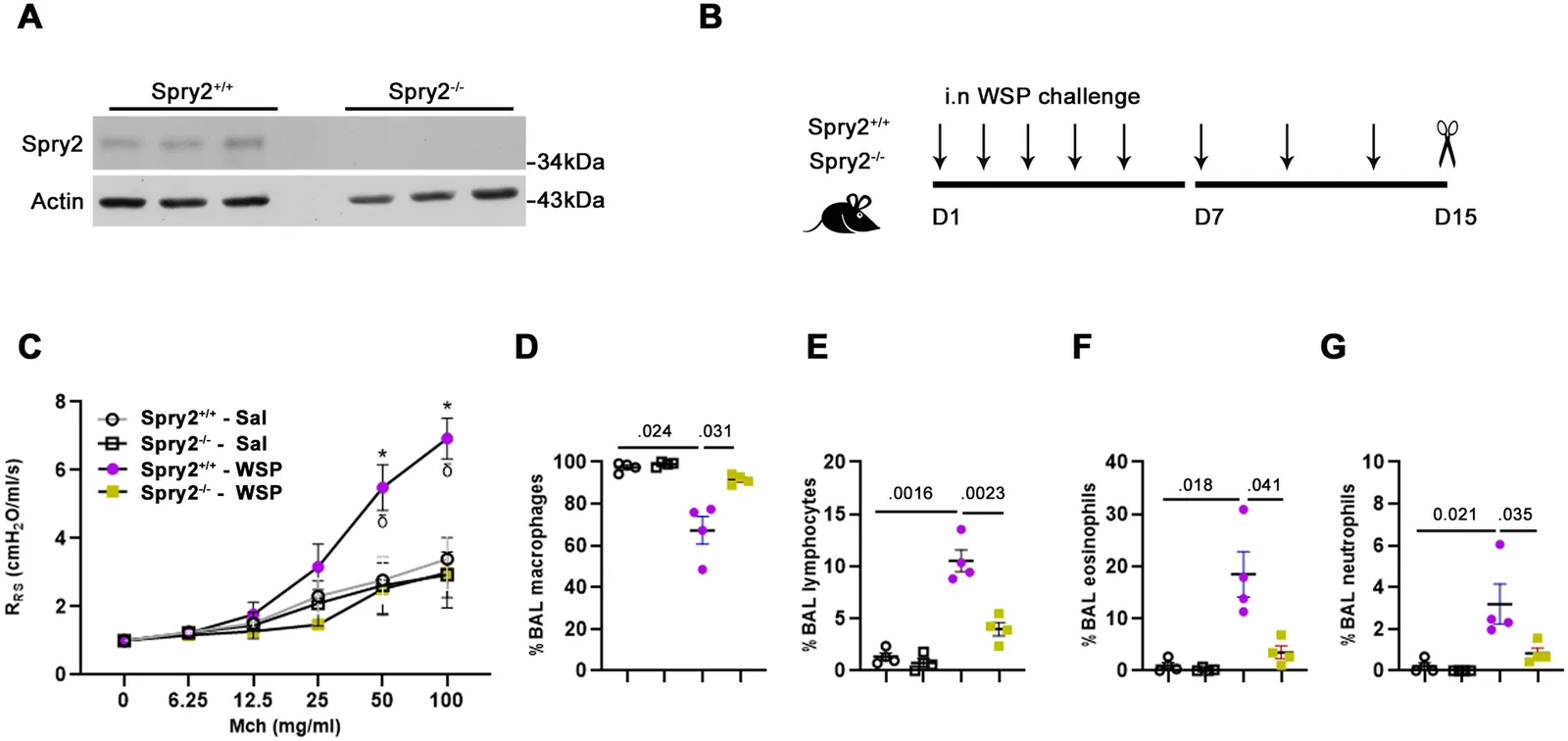
WSP-induced airway hyperreactivity and the role of Spry2. **(A)** Total lung lysates from Tamoxifen-treated *Spry2^f/f^* (*Spry2^+/+^*) (*n* = 3) and ERT2-Cre: *Spry2^f/f^* (*Spry2^−/−^*) mice (*n* = 3) were analyzed by Western blot to confirm Tamoxifen-inducible deletion of Spry2. **(B)** A schematic diagram of the intra nasal (i.n.) administration of Saline or WSP and the experimental end point. **(C)** Airway hyperreactivity (AHR) after Sal or WSP exposure as measured by flexiVent. * *Spry2^+/+^*-WSP versus *Spry2^−/−^*-WSP, *P* < 0.05; *^δ^ Spry2^+/+^*-Sal versus *Spry2^+/+^*-WSP, *P* < 0.05; (*n* = 4). **(D–G)** Differential counts of BAL macrophages, lymphocytes, eosinophils, and neutrophils (*n* = 4). A 2-way ANOVA with Tukey's multiple comparisons test was used to determine the statistical significance between groups. Data are presented as mean ± SEM. *P < 0.05, **P < 0.01.

### Airway hyperreactivity (AHR) measurement

AHR was assessed on a flexiVent v6.2 rodent ventilator (Scireq) as mentioned earlier (21). Briefly, 2 days after the last i.n. challenge, mice were anesthetized using Ketamine (180 mg/kg), xylazine (9 mg/kg), and acepromazine (4 mg/kg). Tracheotomy was performed and mice were attached via an 18-gauge cannula to small-animal ventilator with a computer-controlled piston. AHR measurements were recorded at baseline (Sal) and after increasing concentrations of methacholine (MCh). Resistance measurements were acquired after nebulization of each concentration of MCh. Airway resistance was expressed as total respiratory system resistance. Data are presented as mean ± SEM. Multiple groups were compared using 2-way ANOVA followed by Tukey's *post hoc* multiple comparison test.

### BAL cell differential counts

Recovered BAL fluid was centrifuged (500x*g* for 5 min) and the supernatant was stored at −80 °C. Cell pellet was resuspended in PBS and slides were prepared by cytocentrifugation (Cytospin 3; Shandon Instruments) and stained with Diff Quik (Dade Behring). BAL cell differential counts were determined using morphological criteria under a light microscope with evaluation of ≥500 cells/slide and the differentials were expressed as percent cell number in the BAL fluids.

### Histology and morphometric analysis

Formalin-fixed and paraffin-embedded lung tissue sections (5 μm) were stained with H&E for inflammation, periodic acid–Schiff (PAS) for mucin staining, Masson trichrome staining for collagen deposition and mounted using permount medium (Thermo Fisher Scientific). Images were acquired on a Nikon Eclipse TE2000-U microscope using ×20 dry lenses through a Diagnostics Instruments camera model 4.2 using Spot software 5.0. Inflammation and PAS staining intensity was quantified with Metamorph image acquisition and analysis software (Molecular Devices), as reported previously (22). Airway inflammation was measured as the ratio of the total area of peribronchial and perivascular inflammatory infiltrates over the perimeter of the associated basement membrane obtained from a minimum of 5 airways per mouse and 5 mice per group.

### Single-cell preparation from lung digest

Mouse lungs were processed for single cell preparation as described earlier (23). Briefly, lungs were perfused with PBS and minced in RPMI containing 2% FBS, 50 µg/mL Liberase and 10 µg/mL DNase followed by incubation at 37 °C for 40 min with constant rotation. Enzymatic reaction was stopped by adding ice cold RPMI containing 10% FBS. Lung digests were passed through 70 µm filter to acquire single cell suspension followed by RBC lysis with ACK lysis buffer. Cells were washed twice with 2% FBS containing Hanks' balanced salt solution and immediately used for staining for Flow cytometry and analysis.

### Flow cytometry

For intracellular staining, cells were incubated for 4 h with monensin and brefeldin A (2 μM). Aliquots of 1 × 10^6^ cells were collected and washed with ice-cold phosphate-buffered saline (PBS) followed by staining with a viability dye (Zombie Aqua Fixable Dye, cat. no. 423101) for dead cell exclusion followed by blocking with TruStain FcX™ (Biolegend, #101320) for 15 min at 4 °C. Cells were stained for surface markers: Lineage cocktail (PacificBlue, #133310), Fc*ε*RI*α* (PacificBlue, #134314), Nk1.1 (PacificBlue, #108722) CD45 (FITC, #109806), CD4 (BV711, #100447), CD25 (BV605, #102036), and ST2 (PECy7, #145316) for 30 min at 4 °C followed by intracellular staining for IL5 (PE, #504304), IL13 (PerCP-eFluor710, #46-7133-82), IL17A (APC-Cy7, #06939) and *p*-ERK (Alexa Fluor647, #675504). We used the BD Cytofix/Cytoperm Kit (#554714) for cytokines and the Fixation/Permeabilization Buffer Set from BioLegend (#425401) for *p*-ERK staining. Data were acquired using LSRII (BD Biosciences, San Jose, CA) flow cytometer and analyzed using FlowJo v10 software. Except IL13 (eBioscience, #46-7133-82), all the antibodies used for surface and intracellular staining were purchased from BioLegend, Inc. (San Diego, CA).

### Western blotting

Lung tissue from *Spry2^+/+^* and *Spry2^−/−^* mice was homogenized in RIPA lysis buffer (Thermo fisher scientific, #89900) supplemented with protease inhibitor cocktail (Sigma, #PPC2020). Lung tissue lysates were centrifuged at 10,000 *g* for 10 min at 4 °C and the supernatants were stored at −80 °C. Protein estimation in the tissue lysates was done using BCA protein assay kit (Pierce, #23225) following manufacturer's instructions. Equal amounts of protein were resolved in SDS-PAGE and then transferred onto a nitrocellulose membrane. The blot was blocked in 5% blotto (Santacruz Biotechnology, #sc-2325) made in TBST for 1hr at room temperature (RT) and then incubated overnight with Sprouty2 (CST, cat. no. 14954) or *β*-Actin (CST, #5125). After this the blot was washed 3 times with TBST for 30 min (10 min per wash). The blot was then incubated with appropriate HRP conjugated secondary antibody for 1 h at RT and washed again 3 times with TBST for 30 min. Immunoblots were developed with Pierce ECL reagent (Pierce, #32209) and signal was captured by autoradiography.

### Statistics

Statistical analysis, methods and biological replicates were indicated in the corresponding figure legends. Data analyses were performed using GraphPad Prism 7 software. Data are shown as mean ± SEM. Statistical significance was determined by Tukey's multiple comparison test (for multiple groups). An ANOVA test for multiple comparisons was used to determine the statistical significance among the groups. Data are presented as mean ± SEM, *n* represents biological replicates.

## Results

### Effect of Spry2 deficiency on WSP-induced AHR and BAL inflammatory cell influx

To study the role of Spry2 in WSP-induced airway inflammation, we first generated Spry2 deficient mice (*Spry2^−/−^*) by administering Tamoxifen into 4-wk-old female ERT2-Cre: *Spry2^f/f^* mice. Deletion of Spry2 was confirmed in lung tissue lysates from *Spry2^−/−^* mice (Figure 1A). Tamoxifen treated *Spry2^f/f^* (*Spry2^+/+^*) had normal levels of Spry2 protein which serve as controls in all our experiments. Subsequently, we studied the role of Spry2 in a mouse model of airway inflammation by challenging *Spry2^+/+^* and *Spry2^−/−^* mice intranasally (i.n.) with saline or WSP for 5 consecutive days in the first week and on alternate days in the second week as depicted in Figure 1B. Invasive lung function measurements revealed profound increase in total airway resistance in *Spry2^+/+^* mice challenged with WSP as compared to saline treated *Spry2^+/+^* mice (Figure 1C). However, WSP challenged *Spry2^−/−^* mice displayed a significant decrease in airway resistance relative to WSP treated *Spry2^+/+^* mice (Figure 1C). This dramatic effect of intranasal WSP administration on airway resistance was accompanied by marked suppression of infiltrating lymphocytes, eosinophils and neutrophils in the BAL fluid of WSP treated *Spry2^−/−^* mice as compared to WSP challenged *Spry2^+/+^* mice (Figures 1D–G). Taken together, these results suggest that Spry2 contributes to airway resistance and BAL inflammatory cell influx.

### Effect of Spry2 deficiency on WSP-induced peribronchial and perivascular inflammation

We aimed to further understand the role of Spry2 in WSP-induced airway inflammation by histopathological analysis of formalin-fixed paraffin-embedded lung tissue sections taken from *Spry2^+/+^* and *Spry2^−/−^* mice intranasally (i.n.) challenged with saline or WSP. Repeated administration of WSP resulted in extensive peribronchial and perivascular inflammation in the lung tissue sections of *Spry2^+/+^* mice relative to saline treated mice. However, WSP challenged *Spry2^−/−^* mice, showed significantly lower inflammatory cell accumulation around airways as assessed by H&E staining of lung tissue sections (Figures 2A,B). Similarly, PAS staining revealed increased goblet cell metaplasia in the lung tissue sections of *Spry2^+/+^* mice relative to saline treated mice. In contrast WSP-treated *Spry2^−/−^* mice, exhibited severely abrogated lung goblet cell metaplasia, a signature of reduced mucus production and diminished IL13-induced responses (Figures 2C,D). We also tested whether loss of Spry2 leads to reduced collagen deposition upon repeated WSP exposure. Hence, we stained the lung tissue sections with Masson's trichrome and observed that, similar to saline treated controls, WSP-treated *Spry2^−/−^* mice revealed diminished collagen deposition as compared to WSP-exposed *Spry2^+/+^* mice (Figures 2E,F). These results suggest that Spry2 contributes to WSP-induced peribronchial and perivascular inflammation, mucus production, goblet cell hyperplasia, and collagen deposition.

**Figure 2:**
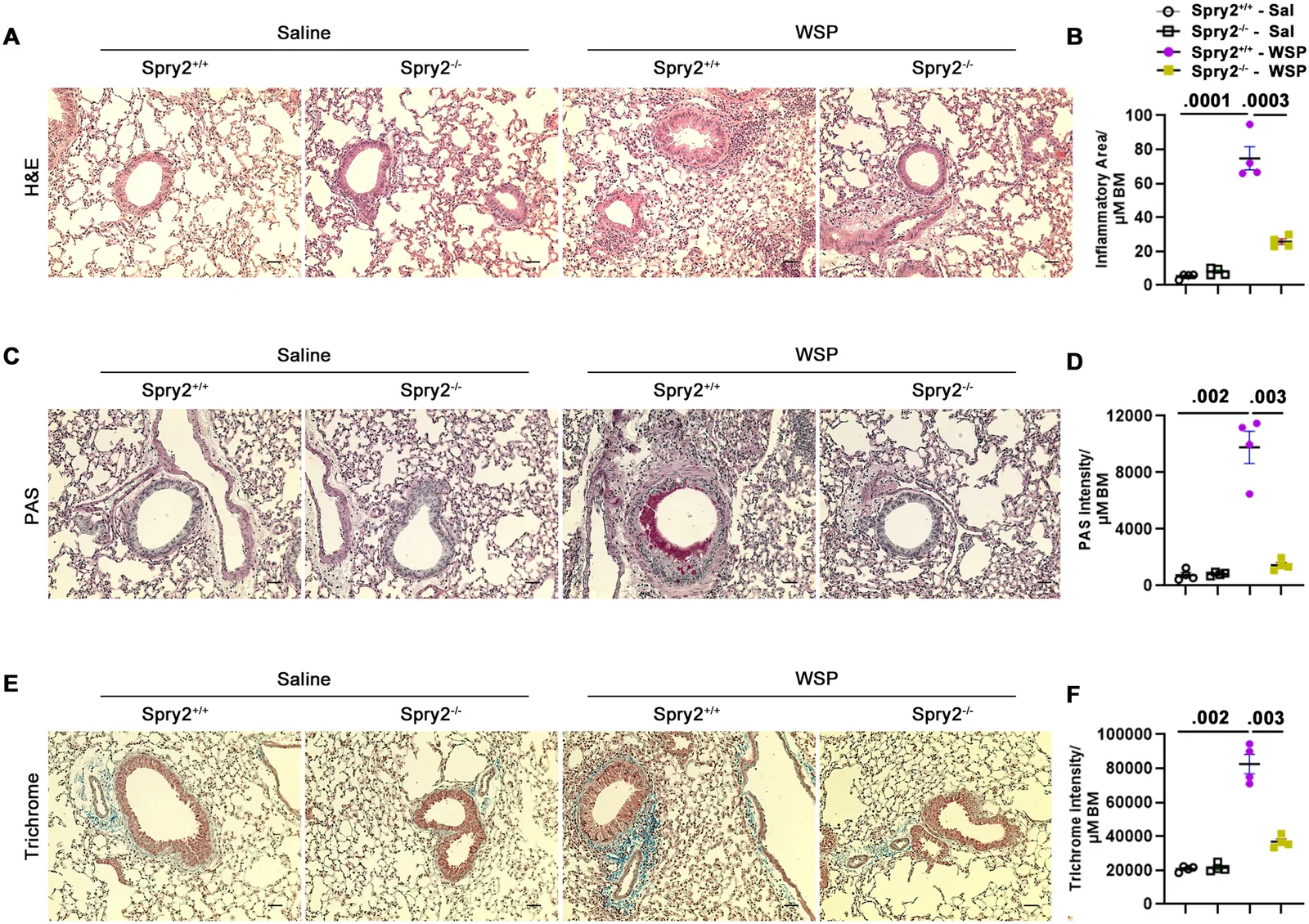
WSP-induced airway pathologies in presence and absence of Spry2. **(A,B)** H&E staining and morphometric quantification of peribronchial and perivascular inflammation per micrometer (μm) of bronchial BM in *Spry2^+/+^* and *Spry2^−/−^* mice treated with Sal or WSP. (*n* = 4). BM, basement membrane; scale bar, 100 µm. **(C,D)** PAS staining and morphometric quantification of epithelial mucus (PAS) staining intensity per micrometer (μm) of bronchial BM in *Spry2^+/+^* and *Spry2^−/−^* mice treated with Sal or WSP. (*n* = 4). BM, basement membrane; Scale bar, 100 µm. **(E,F)** Trichrome staining and morphometric quantification of collagen deposition (green) intensity per micrometer (μm) of bronchial BM in *Spry2^+/+^* and *Spry2^−/−^* mice treated with Sal or WSP. (*n* = 4). BM, basement membrane; Scale bar, 100 µm. A 2-way ANOVA with Tukey's multiple comparisons test was used to determine the statistical significance between groups. Data are presented as mean ± SEM. **P < 0.01, ***P < 0.001.

### Effect of Spry2 deficiency on WSP-induced CD4+ T-cell responses

A recent study has linked smoke exposure with the migration of T cells to inflammatory sites via increased expression of CCR6 (24). We have recently shown the contribution of CD4^+^ T-cell Spry2 to the airway inflammation, AHR, mucous production, and collagen deposition in an allergic airway inflammation model (17). Hence, to test whether Spry2 played a similar role in WSP-induced airway inflammation, we initially performed flow cytometry on single cell suspensions in the lungs of WSP-exposed *Spry2^+/+^* and *Spry2^−/−^* mice. We observed that the percent CD4^+^ T-cells were comparable between the groups exposed to WSP (Figure 3A). We next asked whether ERK signaling is defective in the Spry2 deficient CD4^+^ T-cells. As shown in Figures 3B,C; we observed defective ERK phosphorylation in Spry2 deficient CD4^+^ T-cells relative to Spry2 sufficient CD4^+^ T-cells, exposed to WSP. To test whether this defective ERK phosphorylation leads to reduced IL5, IL13 and IL17A expression, we performed intracellular staining of these cytokines in the lung CD4^+^ T-cells taken from WSP exposed- *Spry2^+/+^* and *Spry2^−/−^* mice. Figures 3D, Supplementary Figure S2A; shows that IL5 expressing CD4^+^ T-cells were comparable between *Spry2^+/+^* and *Spry2^−/−^* mice exposed to WSP. However, we observed significant reduction in IL13 ^+^ and IL17A^+^ CD4^+^ T-cells in Spry2 deficient mice compared to *Spry2^+/+^* mice (Figure 3E, F, Supplementary Figure S2B, C). These results suggest that Spry2 deficiency leads to reduced ERK1/2 phosphorylation, IL13 and IL17A expression, but has no effect on IL5 expression in CD4^+^ T-cells exposed to WSP.

**Figure 3:**
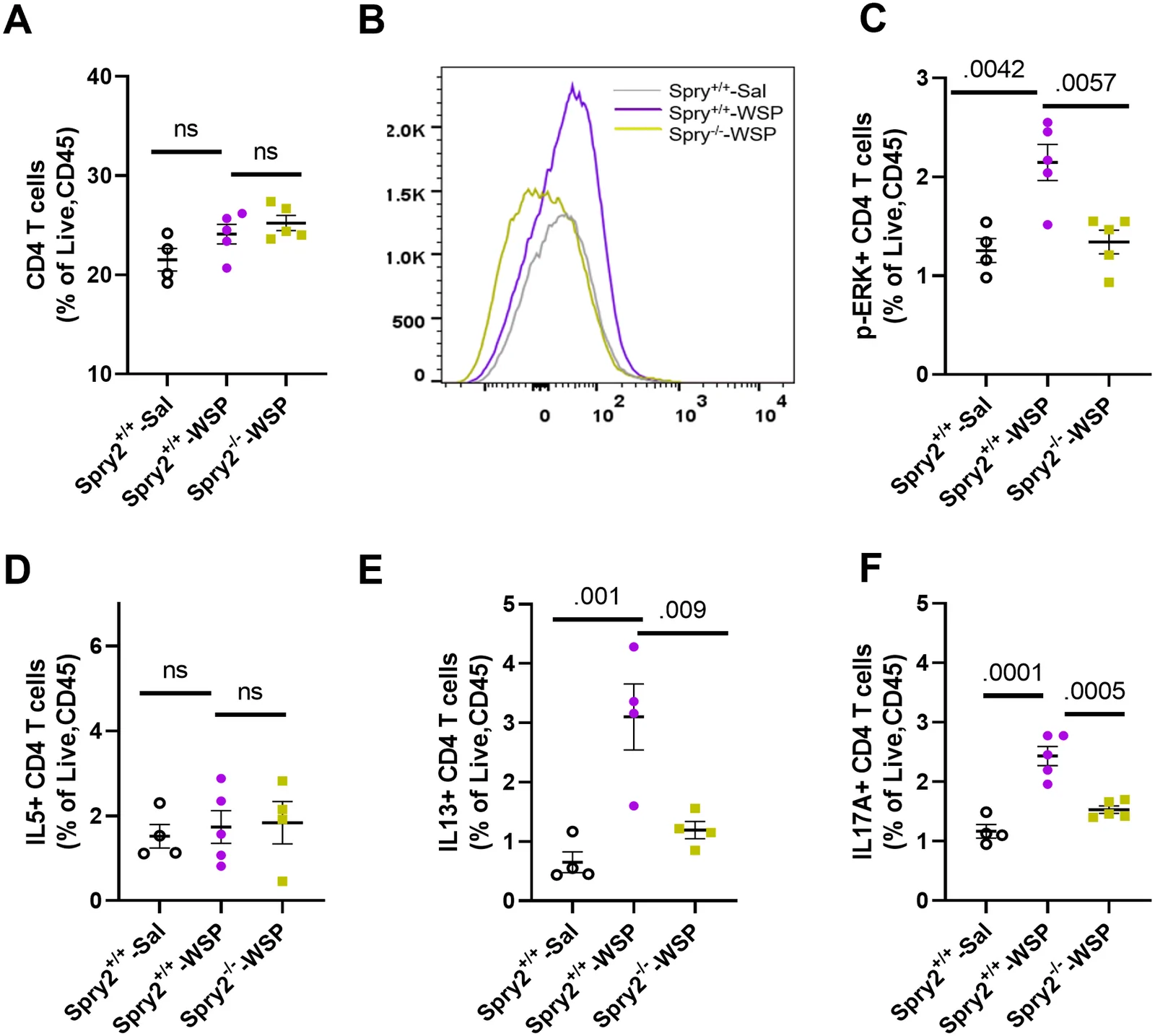
Role of Spry2 in ERK1/2 activation and expression of cytokines by CD4 T cells. **(A)** Statistical analysis of percent lung CD4^+^ T-cells from WSP-administered *Spry2^+/+^* and *Spry2^−/−^* mice (*n* = 5) analyzed by flow cytometry. **(B)** Histogram showing *p*-ERK expression in lung CD4^+^ T-cells from WSP-administered *Spry2^+/+^* and *Spry2^−/−^* mice (*n* = 4). **(C)** Statistical analysis of percent *p*-ERK expressing lung CD4^+^ T-cells from WSP-administered *Spry2^+/+^* and *Spry2^−/−^* mice (*n* = 4) analyzed by flow cytometry. **(D–F)** Statistical analysis of percent intracellular cytokine expressing (IL5^+^, IL13^+^ and IL17A^+^) lung CD4^+^ T-cells from WSP-administered *Spry2^+/+^* and *Spry2^−/−^* mice (*n* = 4) analyzed by flow cytometry. An ANOVA test for multiple comparisons was used to determine the statistical significance among the groups. Data are presented as mean ± SEM, *n* represents biological replicates.

### Effect of Spry2 deficiency on WSP-induced ILC responses

Recent research has shown that innate lymphoid cells (ILCs) contribute to type 2 inflammation by inducing eosinophilia, mucus production and by promoting IL5- and IL13-dependent allergic airway inflammation (25). Hence to test whether Spry2 contributes to WSP-induced ILC responses, we have performed flow cytometry on single cell suspensions in the lungs of WSP-exposed *Spry2^+/+^* and *Spry2^−/−^* mice. Spry2 deficiency led to significant reduction of Lin^−^, CD45^+^, CD25^+^ ILCs relative to Spry2 sufficient mice (Figure 4A). Further, ERK1/2 phosphorylation and percent *p*-ERK expressing ILCs, were also found to be significantly reduced in *Spry2^−/−^* mice as compared to *Spry2^+/+^* mice challenged with WSP (Figures 4B,C). We next asked whether this defective ERK1/2 phosphorylation contributes reduced cytokine expression in *Spry2^−/−^* ILCs. Hence, we performed intracellular staining of IL5, IL13 and IL17 in Lin^−^, CD45^+^, CD25^+^ expressing cells taken from the lungs of WSP exposed *Spry2^+/+^* and *Spry2^−/−^* mice. As shown in Figure 4D–F and Supplementary Figure S3A–C; we observed significant reduction in percent- IL5, IL13 and IL17A expressing ILCs in *Spry2^−/−^* mice relative to *Spry2^+/+^* mice. These results suggest that Spry2 deficiency leads to reduced ERK1/2 phosphorylation, IL5, IL13 and IL17A expression in ILCs exposed to WSP.

**Figure 4:**
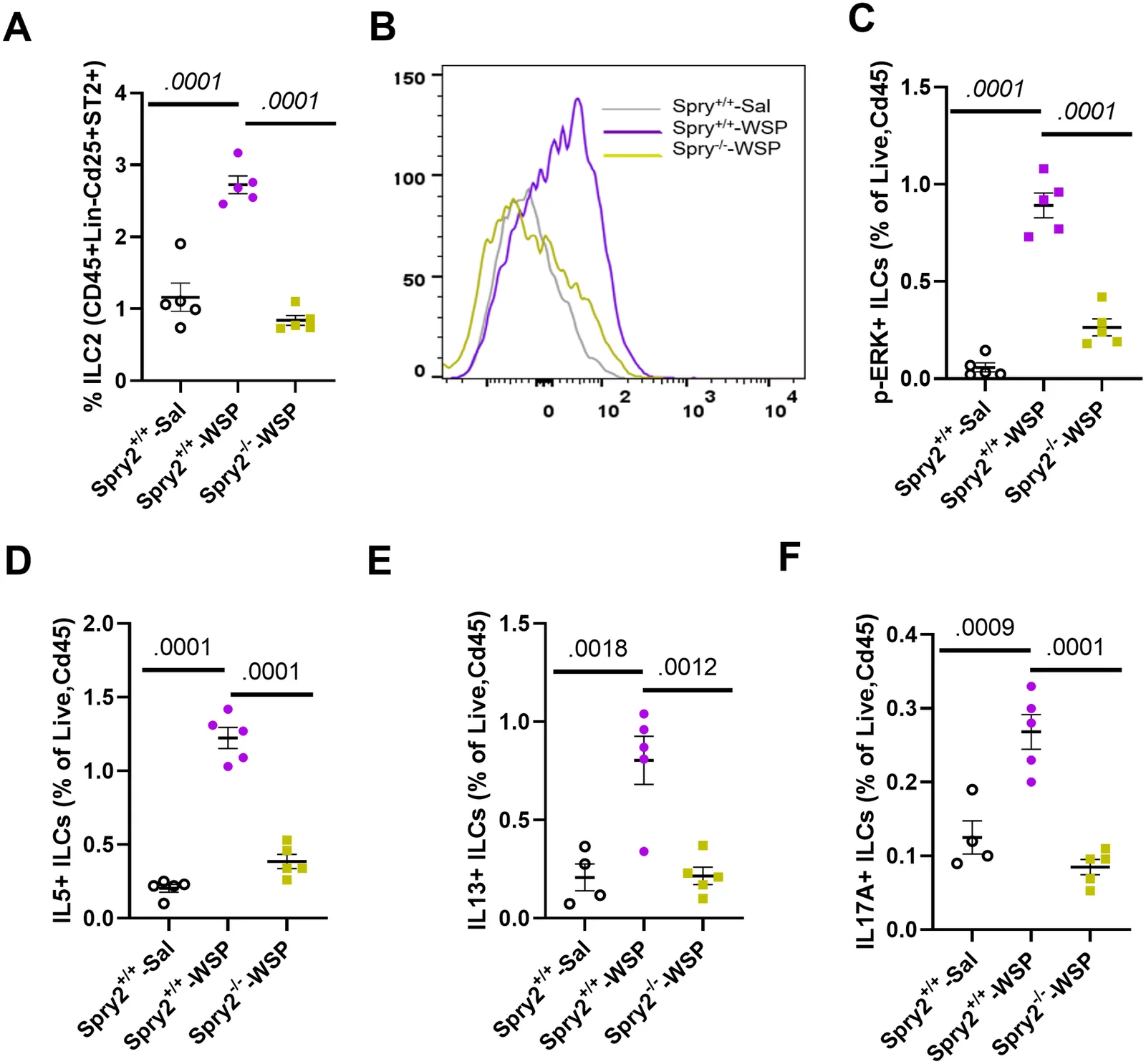
Spry2 regulation of ERK1/2 activation and cytokine expression by ILC2s. **(A)** Statistical analysis of percent lung ILCs from WSP-exposed *Spry2^+/+^* and *Spry2^−/−^* mice (*n* = 5) analyzed by flow cytometry. **(B)** Histogram showing *p*-ERK expression in lung ILCs from WSP-administered *Spry2^+/+^* and *Spry2^−/−^* mice (*n* = 5). **(C)** Statistical analysis of percent *p*-ERK expressing lung ILCs from WSP-administered *Spry2^+/+^* and *Spry2^−/−^* mice (*n* = 5) analyzed by flow cytometry. **(D–F)** Statistical analysis of percent intracellular cytokine expressing (IL5^+^, IL13^+^ and IL17A^+^) lung ILCs from WSP-administered *Spry2^+/+^* and *Spry2^−/−^* mice (*n* = 5) analyzed by flow cytometry. An ANOVA test for multiple comparisons was used to determine the statistical significance among the groups. Data are presented as mean ± SEM, *n* represents biological replicates.

## Discussion

Results presented in this paper demonstrated that a short intranasal exposure of naïve mice to WSP induced airway hyperreactivity, peribronchial and perivascular inflammation with eosinophils and, to a minor extent, neutrophils, mucus production and airway remodeling. This phenotype was dependent on Spry2 signaling as inducible deletion of the Spry2 gene completely abrogated airway hyperreactivity, inflammation, mucus production and airway remodeling. WSP induced activation of ERK1/2 was inhibited in spry2-/- ILC2s and CD4 T cells. Consequently, ILC production of type-2 and type-3 cytokines was inhibited. Our WSP exposure model was designed to favor an innate immune response. For this reason, we observed minimal activation of CD4 T cells. IL17A + but not type-2 cytokine CD4 T cells were inhibited in Spry2-/- mice.

ILC2s are typically activated by receptors for cytokines and endogenous mediators and to a lower extent, pattern recognition receptors (26). IL-24 is induced in epithelial cells by WSP (27) and has been implicated in many pathological states involving inflammation and may play a role in inflammatory bowel disease, psoriasis, cardiovascular disease, rheumatoid arthritis, tuberculosis, and viral infection (28). IL-24 downregulates occludin and ZO-1 expression through the STAT1/STAT3 signaling pathway in epithelial cells, compromising barrier function and increasing permeability (29). The foregoing epithelial abnormalities typically elicit a type-2 immune response. IL-24 promotes IL-33 secretion (30). IL33 is a major activator of ILC2s, Th2 cells and other cells that promote eosinophilic inflammation (31). IL24 activates ERK1/2 and STAT3 to elicit the inflammatory response. IL33, likewise, activates ERK1/2 signaling (32). Spry2 could promote the inflammatory response to WSP-induced IL24 by augmenting ERK1/2 activation. In the absence of spry2, the ERK1/2 activation is impaired, and the allergic phenotype is lost.

Exposure of bronchial epithelial cells to WSP increased the expression of reactive oxygen species and activation of NFkB (33). RNA-seq of WSP-exposed bronchial epithelial cells followed by pathway analysis of the expressed genes showed enrichment in genes belonging to acute phase response, mitochondrial dysfunction, inflammation, oxidative stress, NFkB, ROS, xenobiotic metabolism of aryl hydrocarbon receptor, and chronic respiratory disorder (27). Many of the foregoing pathways such as ROS, oxidative stress, mitochondrial dysfunction and NFkB could activate ILC2s and CD4 T cells and induce a type-2 immune response. Inhalation of particulate matter such as chitin caused increased release of IL33 and TSLP in the airways, which resulted in activation of ILC2s and eosinophilic inflammation in an IRF4-IL9 dependent manner (34).

Note that Spry2 is known as a general inhibitor of receptor tyrosine kinase signaling, especially for inhibiting ERK1/2 signaling. The ERK1/2 inhibition occurs in a cell- and growth factor-dependent manner. The inhibitory activity is most consistent in non-hematopoietic stromal cells, especially when activated by EGF and FGF-type growth factors. However, studies have also demonstrated a potentiating effect of Spry2 on ERK1/2 signaling. Guy and colleagues have shown that Spry2 abrogates EGFR ubiquitylation and endocytosis and sustains EGF-induced ERK signaling in PC12 cells (16). We have reported that Spry2 directly phosphorylates and activates the Src family kinase Fyn, and augments ERK1/2 signaling and activation of lymphoid cells. More recently, we have shown that Spry2 positively regulates NFkB signaling by sequestering PP2A (35). Gupta et al. have shown that WSP-induced ERK1/2 activation in human airway epithelial was inhibited in Spry2 knockdown cells (27). Our gene knockout model has validated this paradigm. Thus, under WSP-stimulating conditions, Spry2 functions as an activator of ERK1/2.

Wood smoke is a common constituent of air pollution worldwide. Wood burning serves as fuel for household cooking and heating in many underdeveloped countries (36). Consequently, WSP is also a common indoor pollution in those countries. The adverse health effect of wildfire and WSP has become a major public health concern (37). Understanding the pathologic consequences of inhaled WSP in the airways is important. Previous studies with firefighters showed respiratory symptoms after a fire-fighting shift, which was associated with increased blood neutrophils, and inflammatory cytokines—IL6, IL8 and MCP1 (CCL2) (38). Exposure of healthy subjects to WSP in an exposure chamber caused an increase in neutrophils in bronchial and bronchoalveolar lavage (39), peripheral blood (39, 40) and induced sputum (40). A small but significant increase in sputum eosinophils in addition to neutrophils was observed in healthy donors upon WSP exposure (41). Another study showed an increase in CC16 in the blood and increased fractional exhaled nitric oxide (FENO) after exposure of healthy subjects to WSP (42). WSP induced barrier dysfunction in the airway epithelial cell layer in an ERK1/2-dependent manner (43) supporting a role of this pathway in epithelial dysfunction. WSP was found to have an adjuvant-like effect on induction of anti-ovalbumin IgE antibody and type-2 inflammation (44).

This study provides immunologic consequences of WSP-driven acute asthma in a mouse model. The results of this study demonstrate that WSP is an inducer of type-2 experimental asthma. WSP has a modest effect on neutrophilic inflammation. Spry2 is a critical regulator of the airway response to WSP. Gupta et al. have identified 52 genetic risk variants that co-localized with genomic responses to WSP (7). One of the risk variants—rs3861144 was associated with asthma (odds ratio = 1.036). rs3861144 changes Spry2 responses to WSP in airway epithelial cells. Thus, subjects with the risk variant rs3861144 are likely to be more susceptible to wildfire smoke and WSP polluted air exposure. In addition to reducing exposures, defining the molecular pathways responsible for the deleterious effects of WSP is critical for developing therapies that directly target this public health threat to further minimize adverse health effects.

## Data Availability

The original contributions presented in the study are included in the article/Supplementary Material, further inquiries can be directed to the corresponding author.

## Limitation of the study

Several limitations should be considered when interpreting these findings. First, we did not directly assess airway epithelial function, which limits our ability to determine the functional consequences of the observed changes. Second, we did not perform targeted knockout (KO) experiments; therefore, the specific causal contribution of various cell types or pathways cannot be definitively established. Third, WSP model was used as a proxy model for wildfire smoke and thus may not fully capture the composition and complexity of real-world wildfire smoke exposure. Finally, the relatively small sample size may limit statistical power and the generalizability of our findings.
