# Supplementary material for: Wood smoke fire particulate matter induces eosinophilic asthma in a Sprouty2-dependent manner in mice
Source: Front Allergy. 2026 Oct 30;7:1951459. doi: 10.3389/falgy.2026.1951459 (PMC13759372; doi:10.3389/falgy.2026.1951459)
Supplement: Supplementary file 1 [file Table1.docx]

Supplementary figures

Supplementary Figure1. Flow cytometric analysis of CD4, ILCs and cytokines. (A-E) The flow cytometry gating strategy for identification of CD4 T (Live, Cd45+, Lin+, CD4+) and ILC2s (CD45+Lin-CD25+ST2+) cell populations in the mouse lung cell digest. (F) FMO controls for p-ERK, IL5, IL13 and Il-17A in CD4 T cell population. (G) FMO controls for p-ERK, IL5, IL13 and Il-17A in ILC2 population.

Supplementary Figure2. Flow cytometric analysis of cytokines in CD4 T cells. Representative dot plots of IL-5 (A), IL13 (B) and IL17A (C) in lung tissues from Spry+/+ and Spry-/- treated with Sal or WSP.

Supplementary Figure3. Flow cytometric analysis of cytokines in ILCs. Representative dot plots of IL-5 (A), IL13 (B) and IL17A (C) in lung tissues from Spry+/+ and Spry-/- treated with Sal or WSP.
